# Loci identified by a genome‐wide association study of carotid artery stenosis in the eMERGE network

**DOI:** 10.1002/gepi.22360

**Published:** 2020-09-22

**Authors:** Melody R. Palmer, Daniel S. Kim, David R. Crosslin, Ian B. Stanaway, Elisabeth A. Rosenthal, David S. Carrell, David J. Cronkite, Adam Gordon, Xiaomeng Du, Yatong K. Li, Marc S. Williams, Chunhua Weng, Qiping Feng, Rongling Li, Sarah A. Pendergrass, Hakon Hakonarson, David Fasel, Sunghwan Sohn, Patrick Sleiman, Samuel K. Handelman, Elizabeth Speliotes, Iftikhar J. Kullo, Eric B. Larson, The eMERGE Consortium, Gail P. Jarvik

**Affiliations:** ^1^ Division of Medical Genetics, School of Medicine University of Washington Seattle Washington USA; ^2^ Department of Biostatistics, Center for Statistical Genetics University of Michigan Ann Arbor Michigan USA; ^3^ Department of Biomedical Informatics and Medical Education, School of Medicine University of Washington Seattle Washington USA; ^4^ Kaiser Permanente Washington Health Research Institute Seattle Washington USA; ^5^ Center for Genetic Medicine Northwestern University Chicago Illinois USA; ^6^ Department of Internal Medicine University of Michigan Ann Arbor Michigan USA; ^7^ Genomic Medicine Institute, Geisinger Danville Pennsylvania USA; ^8^ Department of Biomedical Informatics Columbia University New York New York USA; ^9^ Department of Medicine Vanderbilt University Medical Center Nashville Tennessee USA; ^10^ Division of Genomic Medicine National Human Genome Research Institute Bethesda Maryland USA; ^11^ Geisinger Research Rockville Maryland USA; ^12^ Department of Pediatrics, The Center for Applied Genomics, Children's Hospital of Philadelphia, The Perelman School of Medicine University of Pennsylvania Philadelphia Pennsylvania USA; ^13^ Mayo Clinic Rochester Minnesota USA; ^14^ Department of Pediatrics, The Children's Hospital of Philadelphia, The Perelman School of Medicine University of Pennsylvania Philadelphia Pennsylvania USA; ^15^ Division of Gastroenterology, Department of Internal Medicine and Department of Computational Medicine and Bioinformatics University of Michigan Ann Arbor Michigan USA; ^16^ Department of Cardiovascular Medicine Mayo Clinic Rochester Minnesota USA; ^17^ The electronic Medical Records and GEnomics Network, NHGRI, NIH Bethesda Maryland USA

**Keywords:** carotid artery atherosclerosis, electronic health records, genome‐wide association study

## Abstract

Carotid artery atherosclerotic disease (CAAD) is a risk factor for stroke. We used a genome‐wide association (GWAS) approach to discover genetic variants associated with CAAD in participants in the electronic Medical Records and Genomics (eMERGE) Network. We identified adult CAAD cases with unilateral or bilateral carotid artery stenosis and controls without evidence of stenosis from electronic health records at eight eMERGE sites. We performed GWAS with a model adjusting for age, sex, study site, and genetic principal components of ancestry. In eMERGE we found 1793 CAAD cases and 17,958 controls. Two loci reached genome‐wide significance, on chr6 in LPA (rs10455872, odds ratio [OR] (95% confidence interval [CI]) = 1.50 (1.30–1.73), *p* = 2.1 × 10^−8^) and on chr7, an intergenic single nucleotide variant (SNV; rs6952610, OR (95% CI) = 1.25 (1.16–1.36), *p* = 4.3 × 10^−8^). The chr7 association remained significant in the presence of the LPA SNV as a covariate. The LPA SNV was also associated with coronary heart disease (CHD; 4199 cases and 11,679 controls) in this study (OR (95% CI) = 1.27 (1.13–1.43), *p* = 5 × 10^−5^) but the chr7 SNV was not (OR (95% CI) = 1.03 (0.97–1.09), *p* = .37). Both variants replicated in UK Biobank. Elevated lipoprotein(a) concentrations ([Lp(a)]) and LPA variants associated with elevated [Lp(a)] have previously been associated with CAAD and CHD, including rs10455872. With electronic health record phenotypes in eMERGE and UKB, we replicated a previously known association and identified a novel locus associated with CAAD.

**Abbreviations**
CAAD: carotid artery atherosclerosis disease
CHD: coronary heart disease
CIMT: carotid initima‐media thickness
CPT: current procedural terminology
EHR: electronic health records
eMERGE: electronic Medical Records and Genomics
ICD: international classification of disease
Lp(a): lipoprotein (a) protein
MAF: minor allele frequency
NLP: natural language processing
PCA: principal components analysis
PPV: positive predictive value
SNV: single nucleotide variant
UKB: United Kingdom biobank

## 1 INTRODUCTION

Carotid artery atherosclerotic disease (CAAD) is a major cause of ischemic stroke (Goldstein et al., 2006). Risk factors for CAAD include smoking, dyslipidemia, lipoprotein (a) concentration ([Lp(a)]; Clarke et al., 2009), *LPA* variants (which are associated with [Lp(a)] (Dumitrescu et al., 2011; Ober et al., 2009)), rs1454626 in *VLDLR* (Crawford et al., 2008), and plasma phospholipid transfer protein activity (Clarke et al., 2009). Relatively little work has been done to identify genetic variants associated with CAAD. Many studies have focused on a correlated trait, carotid intima‐media thickness (CIMT; Forgo et al., 2018). The genetics of another correlated condition, coronary heart disease (CHD), have been well characterized in family and association studies (for a review, see, Khera & Kathiresan, 2017).

CHD and carotid atherosclerosis have related environmental and clinical risk factors, but genetic studies suggest different pathophysiologic mechanisms between them. In one study the consequence of carotid atherosclerosis, ischemic stroke, shared only one genetic risk locus out of 25 with CHD (Lövkvist et al., 2013). In another, ischemic stroke and large artery stroke share three and five loci, respectively, with CHD (Dichgans et al., 2014). This may also be the case within carotid artery‐related phenotypes. For example, different loci are associated with two carotid artery phenotypes associated with atherosclerosis, CIMT and carotid plaque (Bis et al., 2011).

The electronic Medical Records and Genomics (eMERGE) Network is a research network that integrates electronic health records (EHR) and genomic data to drive genomic discovery and advance personalized medicine (McCarty et al., 2011). It allows the identification of phenotypes from many participants using existing health data, providing enough cases to power genome‐wide association studies (GWAS). The purpose of the current study was to identify loci associated with EHR‐defined CAAD using the eMERGE GWAS data set. Data from the UK Biobank (UKB) were used to test the robustness of the associations identified in eMERGE.

## 2 MATERIALS AND METHODS

### 2.1 The eMERGE Network

The eMERGE Network is a consortium funded by National Human Genome Research Institute (NHGRI), National Institutes of Health (NIH) since 2007. It includes sites across the United States, with the purpose of linking EHR and genomic data for genomic medicine research. Phenotypes are extracted from EHR by algorithms developed by investigators and applied at each site. It is currently in Phase 3 and includes over 80,000 genotyped participants from nine adults and three pediatric institutions (Chisholm, 2013; Crawford et al., 2014; Gottesman et al., 2013). Enrollment was approved by review boards at each study site and consent obtained, abiding by the Declaration of Helsinki principles.

### 2.2 Phenotype algorithm

We developed the CAAD phenotype algorithm, including a portable natural language processing (NLP) system at Kaiser Permanente Washington/University of Washington (KPWA/UW) using patients from KPWA. Details of the development of the algorithm and its implementation, including a flow diagram are given in the Supplemental Methods. The algorithm extracts the extent of carotid stenosis from imaging reports. We developed it using the approach previously described for creating eMERGE phenotypes (Kho et al., 2012; Kullo et al., 2010). Inclusion criteria included a minimum number of contacts with a patient (in preceding 5 years to most recent record, two records ≥ 1 year apart, or three in different quarters, or four in different months), and minimum age of 18 years. Cases were defined by one or more of the following criteria: more than or equal to 16% carotid artery stenosis (unilaterally or bilaterally) on carotid imaging, which is a standard ultrasound cutoff (Taylor & Strandness, 1987), or at least one International Classification of Disease (ICD)‐9 or Current Procedural Terminology (CPT) code indicating an endarterectomy (ICD‐9 00.63, 38.12, CPT 35301, 35390), or at least two records indicating CAAD (ICD‐9 433.1) more than or equal to 30 days apart. Cases were excluded if they had lab evidence of maximum total cholesterol greater than 400 mg/dl, which would suggest monogenic familial hyperlipidemia, or a pure hypercholesterolemia diagnosis (ICD‐9 272.0). We used this exclusion because for individuals with carotid stenosis and such high cholesterol, their genetic risk is likely driven by hypercholesterolemia‐associated variants. Controls were defined as having an imaging study showing the lowest category of stenosis bilaterally (0%–15% stenosis) or no evidence of carotid artery imaging, no CAAD diagnoses, and no carotid repair procedures. We also collected index age (age of first CAAD diagnosis for cases and age at latest record for controls), sex, self‐reported race and ethnicity, smoking status, CHD defined by at least two ICD‐9 codes for CHD on encounters at least 60 days apart, and body mass index (BMI).

Algorithm evaluation found that 24 out of 25 cases (96% case positive predictive value [PPV]) and all 25 controls (100% control PPV) were validated with chart review at KPWA, and 100% case and control PPV in 20 cases and 20 controls at Marshfield Clinic (Supplemental Methods). We then applied the developed algorithm to adult participants from the Geisinger, Harvard University, KPWA/University of Washington, Marshfield, Mayo, Mt. Sinai Health System, Northwestern, and Vanderbilt University eMERGE sites. Demographics are presented by case status in Table 1, and by site in Table S1.

**Table 1:** Demographics of eMERGE participants included in the CAAD GWAS, separated by CAAD case‐control status

	Case (*N* = 1793)	Control (*N* = 17,958)	Total (*N* = 19,751)
Age (years)			
Mean (*SD*)	71.37 (9.70)	63.45 (16.08)	64.16 (15.77)
Range	29.00–98.00	19.00–106.00	19.00–106.00
Sex			
Female	667 (37.2%)	10390 (57.9%)	11057 (56.0%)
Male	1126 (62.8%)	7568 (42.1%)	8694 (44.0%)
Race^a^			
N‐Miss^b^	5	6	11
African American	61 (3.4%)	4056 (22.6%)	4117 (20.9%)
American Indian/Alaskan Native	4 (0.2%)	48 (0.3%)	52 (0.3%)
Asian	11 (0.6%)	165 (0.9%)	176 (0.9%)
Caucasian	1664 (93.1%)	12,214 (68.0%)	13,878 (70.3%)
Native Hawaiian/Pacific Islander	0 (0.0%)	5 (0.0%)	5 (0.0%)
Not reported	4 (0.2%)	83 (0.5%)	87 (0.4%)
Unknown/Other	44 (2.5%)	1381 (7.7%)	1425 (7.2%)
Ethnicity^a^			
Hispanic/Latino	35 (2.0%)	1192 (6.6%)	1227 (6.2%)
Non Hispanic/Latino	1578 (88.0%)	16,304 (90.8%)	17,882 (90.5%)
Unknown	180 (10.0%)	462 (2.6%)	642 (3.3%)
Ever smoker			
N‐Miss	350	7938	8288
Ever	1134 (78.6%)	5024 (50.1%)	6158 (53.7%)
Never	309 (21.4%)	4996 (49.9%)	5305 (46.3%)
Median BMI (kg/m^2^)			
N‐Miss	279	6394	6673
Mean (*SD*)	28.59 (5.41)	30.08 (7.47)	29.90 (7.28)
Range	17.86–56.98	14.10–80.86	14.10–80.86
Coronary heart disease			
N‐Miss	138	3732	3870
No	631 (38.1%)	11,051 (77.7%)	11,682 (73.6%)
Yes	1024 (61.9%)	3175 (22.3%)	4199 (26.4%)
Site			
Geisinger	591 (33.0%)	1601 (8.9%)	2192 (11.1%)
Harvard	138 (7.7%)	3732 (20.8%)	3870 (19.6%)
Kaiser/University of Washington	227 (12.7%)	2260 (12.6%)	2487 (12.6%)
Marshfield	165 (9.2%)	1920 (10.7%)	2085 (10.6%)
Mayo	407 (22.7%)	235 (1.3%)	642 (3.3%)
Mt. Sinai	82 (4.6%)	4279 (23.8%)	4361 (22.1%)
Northwestern	56 (3.1%)	506 (2.8%)	562 (2.8%)
Vanderbilt	127 (7.1%)	3425 (19.1%)	3552 (18.0%)

Abbreviations: BMI, body mass index; CAAD, Carotid artery atherosclerotic disease; eMERGE, electronic Medical Records and Genomics; GWAS, genome‐wide association studies.

a Self‐reported race and ethnicity.

b Number of missing values.

### 2.3 Genotype data

We imputed 83,717 eMERGE participants’ genotype data to Haplotype Reference Consortium version r1.1 (McCarthy et al., 2016) with the Michigan Imputation Server (MIS; Das et al., 2016), as described previously (Stanaway et al., 2019). Single nucleotide variants (SNVs) were filtered for call rate more than 2% and individuals filtered for genotype missingness less than 2%. Related individuals were identified by calculating identity by descent with the plink2‐genome (Chang et al., 2015) function. Only one person per family was retained, prioritizing cases over controls. We typed participants in the CAAD analysis on one of 24 different chips. For each SNV, we calculated mean imputation quality *R* ^2^ across all chips, using the *R* ^2^ values calculated in the MIS imputation. We also calculated minor allele frequency (MAF) among all CAAD analysis participants and within European and African ancestry subgroups. Principal components analysis (PCA) was performed with plink2 PCA approx method (Chang et al., 2015) on the entire eMERGE cohort, and within European and African ancestry subsets (as described in Stanaway et al, 2019).

### 2.4 Statistical analysis

For the GWAS, we used an additive genotype model and logistic regression in PLINK 1.9 (Chang et al., 2015). We performed analyses in all participants, and in subsets with genetically determined European or African ancestry, identified through the intersection of the k‐means clustering of PC1 and PC2 and the self‐reported or observed ancestry of the participant (Figure S1; Stanaway et al., 2019). Self‐reported Latino/Hispanics were removed from the ancestry stratified analysis. The main analysis included the covariates age, sex, study site, and PC1–10. For the top associated variants, we included BMI, CHD, and smoking status in the model where possible; these were not included in the main analysis because they were not available from all sites. We filtered results shown including only SNVs with MAF more than 0.05 and imputation quality *R* ^2^ > .3. We considered GWAS results statistically significant with a *p*‐value below 5 × 10^−8^, as a correction for multiple testing, and carried those passing that threshold forward for replication in the UK Biobank. Other statistical analyses and plots were done with R (R Core Team 2018).

We performed phenome wide association study (PheWAS; Denny et al., 2010) on the significantly associated SNV that had no previously known phenotypic associations, rs6952610 in the larger eMERGE cohort (*n* = 83,717) to determine if other phenotypes may be associated with this SNP.

### 2.5 UK Biobank

The UK Biobank (UKB) is a population sample of 502,629 participants in the United Kingdom aged 40–69 years at the time of study recruitment (Sudlow et al., 2015). Participants were recruited between 2006 and 2010 from 22 centers across the United Kingdom, in both urban and rural areas, with a broad mixture of socioeconomic backgrounds. All participants provided written, informed consent under each site's local Institutional Review Board. UKB protocols were approved by the National Research Ethics Service Committee, and all ethical regulations were followed. Analyses carried out in UKB specifically for this study were done under approved application 18,120 (Speliotes).

For the UKB, we determined CAAD status via ICD‐10 codes. We specifically assessed for occlusion and stenosis of the carotid artery (ICD‐10 code I65), which includes the subclasses of occlusion and stenosis of the right (I65.21), left (I65.22), bilateral (I65.23), or unspecified (I65.29) carotid arteries. All participants without those codes were included as controls. Due to data limitations, individuals with hypercholesterolemia (max total cholesterol > 400 mg/dl or a coded diagnosis of hypercholesterolemia) were not excluded from the UKB analysis. A total of 934 cases and 408,027 controls had complete genotype, phenotype, and covariate data.

### 2.6 UKB Genotyping and imputation

Participant genotyping, data collection, and quality control has previously been described (Sudlow et al., 2015). In brief, participants were genotyped on either the Affymetrix UK BiLEVE Axiom Array (*N* = 50,520) or the Affymetrix UK Biobank Axiom Array (*N* = 438,692) with 95% overlap between markers on each array. Haplotype phasing of the data was performed with SHAPEIT2 (Delaneau, Howie, Cox, Zagury, & Marchini, 2013; Delaneau, Zagury, & Marchini, 2013). Reference data from the Haplotype Reference Consortium (Loh et al., 2016) was used for imputation (Das et al., 2016).

The largest ancestry group in UKB are self‐identified “White British” that also group closely by genotype PCA (Price, Zaitlen, Reich, & Patterson, 2010). The first 10 principal components in such a large sample will not capture the substructure in all possible ancestries. To increase sensitivity, we included only that group. We filtered genetic variants for a minimum imputation information score of 0.85 and a minimum allele count of 20. Following quality control, 17,981,292 genetic variants in 408,961 participants remained for analysis.

### 2.7 UKB Genome‐wide association analyses

For the GWAS of CAAD in UKB, we used a linear mixed model with saddle point‐correction implemented in the package SAIGE. This approach decreases *p*‐value inflation commonly observed in rare outcome and rare genetic variant associations (Zhou et al., 2018), and is appropriate for the imbalanced case–control ratio in the UKB data. Analyses were adjusted for age, sex, and the first 10 principal components (Price et al., 2006), and the results reported were filtered for MAF more than 0.05.

### 2.8 Annotation of results

We annotated SNVs passing the genome‐wide significance threshold with Genotype‐Tissue Expression project data (GTEx; https://gtexportal.org/home/), European Bioinformatics Institute GWAS catalog (https://ebi.ac.uk/gwas/), and Haploreg (https://pubs.broadinstitute.org/mammals/haploreg/haploreg.php).

## 3 RESULTS

### 3.1 Phenotype and demographics

We identified 1793 cases and 17,958 controls in the eight participating eMERGE sites. Demographics and site of origin for cases versus controls are shown in Table 1. Of the cases, 950 (53.0%) had more than 50% stenosis and/or at least one procedure code for endarterectomy. Of the controls, 411 (2.3%) had an imaging study showing less than or equal to 15% carotid artery stenosis. Participants were 56.0% female, 70.3% self‐reported Caucasian, and 53.7% were ever a smoker.

### 3.2 GWAS results

#### 3.2.1 Pooled Association Results

**Figure 1:**
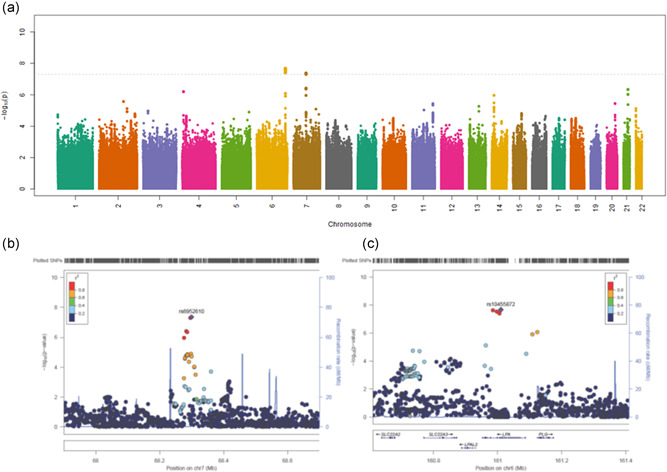
Plots of eMERGE CAAD GWAS results, SNVs arranged by chromosome position and 25 filtered for MAF more than 0.05, imputation quality *R* ^2^ more than .3. (a) Manhattan plot of all result. (b) 26 LocusZoom plot of chromosome 6 locus. (c) LocusZoom plot of choromosome 7 locus. CAAD, Carotid artery atherosclerotic disease; eMERGE, electronic Medical Records and Genomics; GWAS, genome‐wide association studies; SNVs, single nucleotide variants

Two loci were associated with CAAD in the full data set, adjusting for age, sex, eMERGE site, and the first 10 ancestry principal components, and with a minimum *p*‐value of 5 × 10^−8^ (Table 2). These were a locus in the 25th intron of *LPA* (lead SNV rs10455872), and an intergenic region on chromosome 7 between *TYW1* and *AUTS2* (lead SNV rs6952610; Table 2, Figure 1). *LPA* variant rs10455872 is a GTEx eQTL for increased expression of *SLC22A3* in “Skin – Sun Exposed (Lower leg)” (*p* = 2 × 10^−6^), and alters four predicted transcription factor binding motifs: Foxd1_1, Foxf1, Foxj1_2, and Foxo_1. It has entries for association with 16 traits in the GWAS catalog, mostly cardiovascular and lipid phenotypes. Intergenic variant rs6952610 is not a GTEx eQTL, and has no associations in the GWAS catalog. It changes two predicted transcription factor binding motifs: GR_disc1 and GR_known3 (from Haploreg; Ward & Kellis, 2012), data from ENCODE (ENCODE Project Consortium, 2012).

**Table 2:** SNVs with *p* < 5 × 10^−8^ in the eMERGE CAAD GWAS with covariates age, sex, study site, and first 10 principal components

**rsID**	**Chr**	**Position (hg19)**	**A1** a	**A2** a	**OR (95% CI)**	***p***	**MAF**	**MAF EUR** b	**MAF AFR** b	**Mean *R*^2^** c	***N* chips genotyped** d
rs118039278	6	160985526	A	G	1.49 (1.3–1.72)	2.32e−08	0.0535	0.067	0.0115	0.871	0
rs74617384	6	160997118	T	A	1.49 (1.29–1.72)	2.91e−08	0.0535	0.067	0.0115	0.864	0
rs55730499	6	161005610	T	C	1.48 (1.29–1.7)	3.9e−08	0.0546	0.069	0.0117	0.855	0
rs10455872	6	161010118	G	A	1.5 (1.3–1.73)	2.12e−08	0.0533	0.067	0.0117	0.858	3
rs7792316	7	68296349	A	G	1.25 (1.15–1.36)	4.91e−08	0.433	0.36	0.683	0.966	1
rs6952610	7	68299862	T	C	1.25 (1.16–1.36)	4.27e−08	0.429	0.36	0.669	0.964	0

Abbreviations: CAAD, Carotid artery atherosclerotic disease; CI, confidence interval; eMERGE, electronic Medical Records and Genomics; GWAS, genome‐wide association studies; SNVs, single nucleotide variants; OR, odds ratio.

a A1 is the effect and minor allele, A2 the reference allele.

b Minor allele frequency (MAF) for EUR =  European ancestry, AFR = African ancestry subsets, as defined by *k*‐means clustering of genotypes.

c Mean imputation quality *R* ^2^ for that variant.

d Number of chips of 24 total where the SNV was typed rather than imputed.

#### 3.2.2 Associations through ancestry stratification

We defined ancestry groups via *k*‐means clustering of genetic markers only, as many participants were missing self‐reported ancestry. The *LPA* association remained statistically significant in the European ancestry subset (1705 cases, 13,198 controls; *p* = 1.52 × 10^−8^) but the *TYW1*–*AUTS2* association did not reach genome‐wide significance (*p* = 7.45 × 10^−7^). Only 89 (5.0%) cases were of non‐European ancestry, 70 of those were of genetically determined African ancestry. As expected, given the limited power, we found no significant associations in the African ancestry subset (results not shown).

#### 3.2.3 Other covariates

The addition of covariates other than age, sex, site, and PC1–10 did not substantially change the results. We individually added BMI, CHD, and smoking to the logistic model, as well as combining all covariates in a single model (Table 3). Sample sizes differed in these analyses due to missing data. When we added an *LPA* SNV (rs118039278) as a covariate, the *TYW1*–*AUTS2* SNV rs6952610 remained associated (*p* = 4.3 × 10^−8^).

**Table 3:** Secondary analyses of two lead SNVs from eMERGE CAAD GWAS, rs10455872 and rs6952610, with additional covariates added to the main, and a combined model including all

Covariate	OR (95% CI)	*p*	*N* cases	*N* controls
rs10455872*LPA*				
Age, sex, site, PC1–10 (main)	1.5 (1.3–1.73)	2.12e−8	1793	17,958
Main + BMI	1.5 (1.3–1.73)	4.01e−08	1746	17,072
Main + CHD	1.39 (1.19–1.63)	3.27e−05	1655	14,223
Main + ever smoker	1.44 (1.23–1.7)	1.19e−05	1443	10,018
Main + BMI, CHD, ever smoker	1.4 (1.17–1.67)	.000169	1417	9703
rs6952610 TYW1–AUTS2				
Age, sex, site, PC1–10 (main)	1.25 (1.16–1.36)	4.27e−08	1793	17,958
Main + BMI	1.27 (1.17–1.38)	1.37e−08	1746	17,072
Main + CHD	1.27 (1.17–1.39)	5.08e−08	1655	14,223
Main + ever smoker	1.26 (1.15–1.38)	8.42e−07	1443	10,018
Main + BMI, CHD, ever smoker	1.29 (1.17–1.42)	2.19e−07	1417	9703

Abbreviations: BMI, body mass index; CAAD, Carotid artery atherosclerotic disease; CHD, coronary heart disease; CI, confidence interval; eMERGE, electronic Medical Records and Genomics; GWAS, genome‐wide association studies; OR, odds ratio; SNVs, single nucleotide variants.

#### 3.2.4 Coronary heart disease

We also evaluated the association of CHD with the putative CAAD loci in the same data set. There were 4199 cases and 11,684 controls, defined by the presence of a CHD diagnosis. The *LPA* variant was significantly associated with CHD, (OR (95% CI) = 1.27 (1.13–1.43), *p* = 5.5 × 10^−5^), whereas the intergenic chr7 SNV was not (OR (95% CI) = 1.03 (0.97–1.09), *p* = .30).

#### 3.2.5 Replication in the UKB

We tested the six SNVs with *p* < 5 × 10^−8^ in our main analysis in the UKB (Table 4). The *LPA* association replicated (rs10455872 *p* = 3.4 × 10^−6^), as did the chr7 SNV (rs6952610 *p* = .0096). Both were statistically significant at the 5% level, after adjustment for the two contrasts.

**Table 4:** Association of significant eMERGE SNVs with CAAD in the UK Biobank

rsID	Chr	Position (hg19)	eMERGE *p*	UKB *p*	UKB *β*	UKB *SE*	UKB MAF	eMERGE MAF
rs118039278	6	160985526	2.32e−08	2.61e−06	.41	0.088	0.081	0.054
rs74617384	6	160997118	2.91e−08	3.04e−06	.41	0.088	0.081	0.054
rs55730499	6	161005610	3.9e−08	3.87e−06	.40	0.087	0.081	0.055
rs10455872	6	161010118	2.12e−08	3.43e−06	.41	0.087	0.081	0.053
rs7792316	7	68296349	4.91e−08	.00792	.12	0.048	0.37	0.43
rs6952610	7	68299862	4.27e−08	.00962	.13	0.048	0.37	0.43

Abbreviations: CAAD, Carotid artery atherosclerotic disease; eMERGE, electronic Medical Records and Genomics; MAF, minor allele frequency; SNVs, single nucleotide variants.

#### 3.2.6 GWAS in the UKB

As a secondary analysis, we performed a GWAS within the UKB with the ICD‐10 defined phenotype. Two loci reach genome‐wide significance, on chromosomes 1 and 7 (see Figure S2, Manhattan plot and Table S2 of the most significant results). The lead SNV on chr7 was rs2526620, which is intergenic between *HDAC9* and *TWIST1*, and is not near the chr7 SNV in the eMERGE GWAS. The lead SNV on chromosome 1, rs682112, is in an intron of neuron navigator 1 (*NAV1*). *NAV1* is expressed more in the aorta than other GTEx tissues (gtexportal. org). rs682112 is in the GTEx catalog as an eQTL for *NAV1* in Heart—Atrial Appendage (normalized effect size = 0.22, *p* = 5.6 × 10^−8^) and Esophagus—Mucosa (normalized effect size = −0.18, *p* = 2.9 × 10^−5^). These did not replicate in the eMERGE data.

## 4 DISCUSSION

### 4.1 GWAS results

We found two loci, tagged by rs10455872 and rs6952610, associated with CAAD in the eMERGE Network participants. Both replicated in UKB with a simple ICD‐only phenotype, despite differences in the patient demographics and phenotype definition. UKB is a younger, population‐based cohort with less depth of longitudinal data than most eMERGE sites (Sudlow et al., 2015). Longitudinal data can result in deeper phenotyping for participants, allowing improved assignment of case–control status. In addition, no NLP or image analysis was possible in UKB. Due to these differences, proportionally more cases were found in eMERGE: 9.1% in eMERGE versus 0.23% in UKB. Even when comparing the more severe cases, eMERGE cases with more than or equal to 50% stenosis and/or at least one endarterectomy, there were more than UKB: 950, or 4.8% of participants. In KPWA, where validation was performed, 76 of 300 cases were included due to the ICD‐9 code 433.1; the rest had imaging studies or endarterectomy.

Both SNVs were associated in the eMERGE all‐participant analysis and the European ancestry subset, although the chr7 SNV did not pass genome‐wide significance in European ancestry alone. Other ancestry subsets were too small for subgroup analyses. The [Lp(a)]‐associated risk for CAAD varies by race/ethnicity; the burden of risk is higher in individuals with European than African ancestry, but still unclear in Hispanic and Asian populations (Steffen et al., 2019). More work on the risk and genetic predictors of CAAD in non‐European ancestry groups is warranted.

The addition of BMI as a covariate had little impact on the association of either locus. Adding smoking and CHD to the model altered effect sizes and *p*‐values; however, these results are difficult to compare, primarily due to the large drop in sample size due to missing data, as well as due to possible pleiotropic effects of *LPA* variation on both CHD and CAAD. The lead *LPA* SNV was also associated with CHD, but the chr7 SNV rs6952610 was not. Based on the current study and others (Bis et al., 2011; Dichgans et al., 2014; Lövkvist et al., 2013), these phenotypes appear to have overlapping, but also distinct genetic risk factors.

### 4.2 LPA variant

*LPA* variant rs10455872 has been shown to explain 17% of the variance in [Lp(a)] (Ronald et al., 2011). Lp(a) is a small low‐density lipoprotein (LDL)‐like particle formed by apolipoprotein B (apoB) covalently bound to apolipoprotein(a). Rs10455872 is associated with CHD (Nelson et al., 2017), large artery stroke and ischemic stroke (Dichgans et al., 2014), and myocardial infarction (Nikpay et al., 2015). In addition, rs10455872‐G is associated with CHD events independent of statin‐induced lowering of LDL‐C (Khera et al., 2014; Wei et al., 2018), and with smaller LDL‐C reduction with rosuvastatin (Chasman et al., 2012). Other *LPA* variants that predict elevated [Lp(a)] have previously been shown to be associated with CAAD (Ronald et al., 2011). The minor alleles of rs10455872 mark haplotypes carrying short KIV2 kringle repeat alleles that are associated with elevated plasma [Lp(a)] (Ronald et al., 2011), due to increased liver secretion. The KIV4 repeat has been shown to account for 69% of [Lp(a)] plasma variation (Emdin et al., 2016); thus, we expect that rs10455872 predicts both [Lp(a)] and CAAD due to its association with KIV4 repeat length.

Measured and genetically predicted [Lp(a)] is associated with CHD risk (Emdin et al., 2016), but [Lp(a)] is not often measured clinically (Burgess et al., 2018), in part because it is not very responsive to current treatments. People with high [Lp(a)] can have their risk managed with other approaches, and, may benefit from new drugs being developed (Nordestgaard, Nicholls, Langsted, Ray, & Tybjærg‐Hansen, 2018). Burgess et al. (2018) analyzed CHD risk based on genetically predicted Lp(a) and concluded that pharmacological lowering of [Lp(a)] by 100 mg/dl would reduce risk of CHD by 22%–25% in a 3–5 year trial (Burgess et al., 2018). Our results suggest an etiological relationship between elevated [Lp(a)] and CAAD; thus, lowering [Lp(a)] also has the potential to reduce CAAD risk. These data could be used for prevention efforts, including constructing polygenic risk scores.

### 4.3 TYW1–AUTS2

Unlike *LPA*, the chr7 intergenic locus does not have an obvious nearby candidate gene or regulatory epigenetic role, other than altering predicted transcription factor binding motifs of unknown relevance. This SNV is intergenic between *TYW1*–*AUTS2*. TRNA‐YW synthesizing protein 1 homolog (*TYW1)* codes for a protein that stabilizes codon–anticodon interactions in the ribosome. *AUTS2*, autism susceptibility candidate 2, is an activator of transcription, has been shown to regulate neuronal migration and have a role in brain development (Hori & Hoshino, 2017; Sultana et al., 2002). Variation in this gene has been associated with intellectual disability, microcephaly, autism, and other behavioral phenotypes (Beunders et al., 2016). A PheWAS of rs6952610 in a larger segment of eMERGE (*N* = 83,717) yielded no phenome‐wide significant results. However, the top four phecodes were “Musculoskeletal symptoms referable to limbs,” “Other disorders of middle ear and mastoid,” “Nephritis and nephropathy with pathological lesion,” and “Cerebral ischemia,” which is a condition caused by insufficient blood flow to the brain. The later result may suggest cerebral ischemia due to CAAD; however, this would require further evidence as it is not statistically significant when the multiple tests are considered. See Figure S3, the Phewas Manhattan plot and Table S3 of the top Phewas results.

### 4.4 Use of natural language processing

The portable NLP system's built‐in support for local evaluation of NLP performance, combined with centralized modification and re‐distribution of site‐tailored versions of the NLP system, allowed for accurate extraction of quantitative stenosis severity measures from heterogeneously sourced free‐text imaging reports, a known challenge in multisite application of clinical NLP (Carrell et al., 2017).

### 4.5 Limitations

Electronic health records provide an efficient way to identify cases and controls. However, people with no evidence of CAAD in their medical record may have subclinical carotid atherosclerosis, but would be included as controls. This would reduce the power and thus the effect size we see from true associations. As [Lp(a)] is not often clinically measured, we do not have it for these participants. Therefore, we were unable to show that inclusion of this phenotype would eliminate the effect of the genotype, as has been previously reported (Ronald et al., 2011). As discussed above, other limitations were the different phenotype definitions in eMERGE and UKB and the lack of ancestral diversity in these cohorts.

### 4.6 Conclusions

In summary, we identified and replicated two loci associated with CAAD, a risk factor for stroke. The *LPA* association validates prior work that *LPA* variation which affects [Lp(a)] is associated with carotid artery disease and suggests [Lp(a)] is an important therapeutic target for CAAD. The second association, on chr7, is novel and its mechanism of action is unknown.

## CONFLICT OF INTERESTS

The authors declare that there are no conflict of interests.

## AUTHOR CONTRIBUTIONS

The following authors made substantial contributions to conception and design, or acquisition of data, or analysis and interpretation of data; were involved in drafting the manuscript or revising it critically for important intellectual content: David R. Crosslin, Elisabeth A. Rosenthal, David S. Carrell, David J. Cronkite, Adam Gordon, Xiaomeng Du, Yatong K. Li, Marc S. Williams, Chunhua Weng, Qiping Feng, Rongling Li, Sarah A. Pendergrass, Hakon Hakonarson, David Fasel, Sunghwan Sohn, Patrick Sleiman, Samuel K. Handelman, Elizabeth Speliotes, Iftikhar J. Kullo, and Eric B. Larson. In addition to the above, the following authors also gave final approval of the version to be published, Melody R. Palmer, Daniel Seung Kim, Ian B. Stanaway, and Gail P. Jarvik.

## Supporting information

Supporting information.

## Data Availability

These data have been posted to dbGaP, study ID phs000888.v1.p1. This study ID contains the eMERGE genotyped and imputed SNV data. The CAAD phenotype can be found with variable accession number phv00225976.v1.p1.
