# Supplementary material for: Loci identified by a genome‐wide association study of carotid artery stenosis in the eMERGE network
Source: Genet Epidemiol. 2020 Sep 22;45(1):4–15. doi: 10.1002/gepi.22360 (PMC7891640; doi:10.1002/gepi.22360)
Supplement: Supplementary file 1 — Supporting information. [file GEPI-45-4-s001.docx]

eMERGE CAAD Supplemental Information

Table of Contents

Supplemental Results 2

Table S1. Demographics of eMERGE participants included in the CAAD GWAS, separated by site 2

Figure S1. PC 1 & 2 colored by self-reported race 4

Table S2. Lead SNVs from UK Biobank CAAD GWAS. 5

Figure S2. Manhattan plot for UKB GWAS 5

Table S3. Lowest p-val: Musculoskeletal symptoms referable to limbs symptoms 6

Figure S3. PheWAS of rs6952610 in eMERGE 6

Supplemental Methods 7

CAAD Phenotype Algorithm Pseudo Code 7

1. Introduction: 7

2. Development 7

3. Data Requirements 7

4. General Implementation Steps 8

5. Using the NLP System 8

6. General Inclusion Criteria 9

7. CAAD Case Criteria 10

8. CAAD Control Criteria 11

9. KPWA/UW Counts of Cases, Controls, and Excluded Patients 12

10. Data Dictionary and Covariates 13

11. Flow diagram 14

# Supplemental Results

## Table S1. Demographics of eMERGE participants included in the CAAD GWAS, separated by site

|  | Geisinger  (N=2192) | Harvard  (N=3870) | Kaiser/  University of Washington  (N=2487) | Marshfield  (N=2085) | Mayo  (N=642) | Mt. Sinai  (N=4361) | Northwestern (N=562) | Vanderbilt (N=3552) | Total (N=19751) |
| --- | --- | --- | --- | --- | --- | --- | --- | --- | --- |
| **CAAD status** |  |  |  |  |  |  |  |  |  |
| Case | 591 (27.0%) | 138 (3.6%) | 227 (9.1%) | 165 (7.9%) | 407 (63.4%) | 82 (1.9%) | 56 (10.0%) | 127 (3.6%) | 1793 (9.1%) |
| Control | 1601 (73.0%) | 3732 (96.4%) | 2260 (90.9%) | 1920 (92.1%) | 235 (36.6%) | 4279 (98.1%) | 506 (90.0%) | 3425 (96.4%) | 17958 (90.9%) |
| **Age** (years) |  |  |  |  |  |  |  |  |  |
| Mean (SD) | 65.44 (14.28) | 56.08 (16.61) | 81.52 (9.89) | 70.08 (10.11) | 71.33 (10.60) | 60.48 (13.79) | 55.52 (15.03) | 61.17 (13.75) | 64.16 (15.77) |
| Range | 23.00 - 89.00 | 19.00 - 96.00 | 45.00 - 106.00 | 32.00 - 86.00 | 23.00 - 95.00 | 30.00 - 94.00 | 25.00 - 97.00 | 29.00 - 100.00 | 19.00 - 106.00 |
| **Sex** |  |  |  |  |  |  |  |  |  |
| Female | 1034 (47.2%) | 2276 (58.8%) | 1375 (55.3%) | 1276 (61.2%) | 227 (35.4%) | 2514 (57.6%) | 464 (82.6%) | 1891 (53.2%) | 11057 (56.0%) |
| Male | 1158 (52.8%) | 1594 (41.2%) | 1112 (44.7%) | 809 (38.8%) | 415 (64.6%) | 1847 (42.4%) | 98 (17.4%) | 1661 (46.8%) | 8694 (44.0%) |
| **Race*^a^*** |  |  |  |  |  |  |  |  |  |
| N-Miss*^b^* | 0 | 0 | 0 | 0 | 11 | 0 | 0 | 0 | 11 |
| African American | 6 (0.3%) | 211 (5.5%) | 73 (2.9%) | 1 (0.0%) | 1 (0.2%) | 2771 (63.5%) | 89 (15.8%) | 965 (27.2%) | 4117 (20.9%) |
| American Indian/Alaskan Native | 2 (0.1%) | 0 (0.0%) | 11 (0.4%) | 23 (1.1%) | 1 (0.2%) | 11 (0.3%) | 0 (0.0%) | 4 (0.1%) | 52 (0.3%) |
| Asian | 0 (0.0%) | 65 (1.7%) | 71 (2.9%) | 12 (0.6%) | 0 (0.0%) | 2 (0.0%) | 0 (0.0%) | 26 (0.7%) | 176 (0.9%) |
| Caucasian | 2183 (99.6%) | 3278 (84.7%) | 2231 (89.7%) | 2038 (97.7%) | 623 (98.7%) | 512 (11.7%) | 472 (84.0%) | 2541 (71.5%) | 13878 (70.3%) |
| Native Hawaiian/Pacific Islander | 0 (0.0%) | 0 (0.0%) | 5 (0.2%) | 0 (0.0%) | 0 (0.0%) | 0 (0.0%) | 0 (0.0%) | 0 (0.0%) | 5 (0.0%) |
| Not reported | 0 (0.0%) | 0 (0.0%) | 87 (3.5%) | 0 (0.0%) | 0 (0.0%) | 0 (0.0%) | 0 (0.0%) | 0 (0.0%) | 87 (0.4%) |
| Unknown/Other | 1 (0.0%) | 316 (8.2%) | 9 (0.4%) | 11 (0.5%) | 6 (1.0%) | 1065 (24.4%) | 1 (0.2%) | 16 (0.5%) | 1425 (7.2%) |
| **Ethnicity*^a^*** |  |  |  |  |  |  |  |  |  |
| Hispanic/Latino | 10 (0.5%) | 203 (5.2%) | 51 (2.1%) | 11 (0.5%) | 3 (0.5%) | 924 (21.2%) | 0 (0.0%) | 25 (0.7%) | 1227 (6.2%) |
| Non Hispanic/Latino | 1930 (88.0%) | 3667 (94.8%) | 2373 (95.4%) | 2074 (99.5%) | 476 (74.1%) | 3283 (75.3%) | 557 (99.1%) | 3522 (99.2%) | 17882 (90.5%) |
| Unknown | 252 (11.5%) | 0 (0.0%) | 63 (2.5%) | 0 (0.0%) | 163 (25.4%) | 154 (3.5%) | 5 (0.9%) | 5 (0.1%) | 642 (3.3%) |
| **Ever smoker** |  |  |  |  |  |  |  |  |  |
| N-Miss | 11 | 3870 | 495 | 2 | 89 | 269 | 0 | 3552 | 8288 |
| Ever | 1488 (68.2%) | 0 (NaN%) | 818 (41.1%) | 1097 (52.7%) | 480 (86.8%) | 1984 (48.5%) | 291 (51.8%) | 0 (NaN%) | 6158 (53.7%) |
| Never | 693 (31.8%) | 0 (NaN%) | 1174 (58.9%) | 986 (47.3%) | 73 (13.2%) | 2108 (51.5%) | 271 (48.2%) | 0 (NaN%) | 5305 (46.3%) |
| **Median BMI (kg/m^2))** |  |  |  |  |  |  |  |  |  |
| N-Miss | 7 | 3870 | 679 | 1865 | 15 | 163 | 12 | 62 | 6673 |
| Mean (SD) | 32.94 (8.54) | NA (NA) | 26.58 (4.90) | 29.28 (5.73) | 28.94 (4.96) | 30.26 (7.39) | 28.29 (7.30) | 29.76 (6.90) | 29.90 (7.28) |
| Range | 16.99 - 80.86 | NA - NA | 14.80 - 57.50 | 18.69 - 56.81 | 17.46 - 51.94 | 15.29 - 68.06 | 15.90 - 59.91 | 14.10 - 70.72 | 14.10 - 80.86 |

1. Self-reported race and ethnicity
2. Number of missing values

## Figure S1. PC 1 & 2 colored by self-reported race


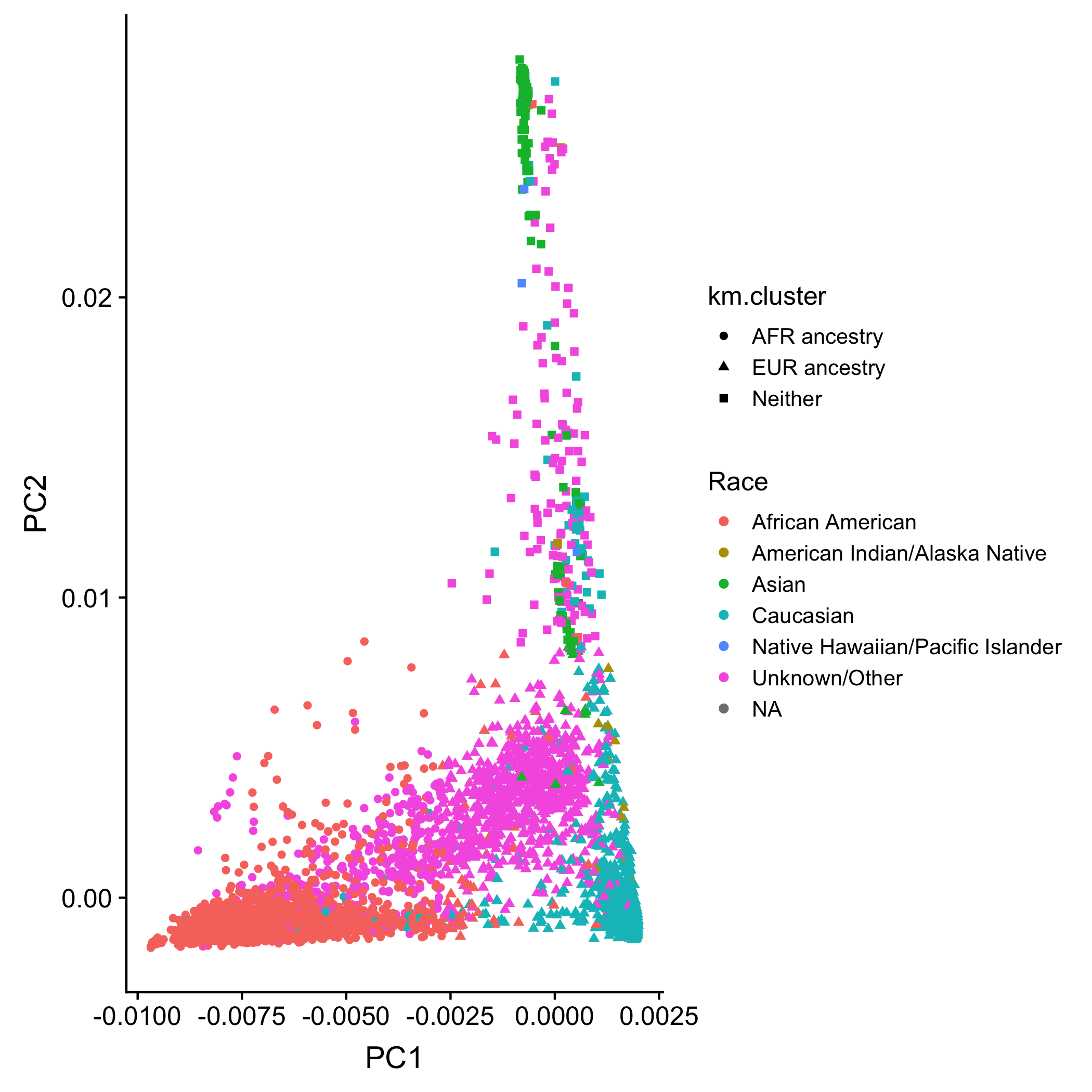


Mt. Sinai had the most cases with African ancestry: 41 cases and 3,068 controls based on k-means clustering – including 10 people self-identified as American Indian/Alaska Native, 3 as Caucasian, and 372 as Unknown or Other.

4540 individuals were removed for relatedness

## Figure S2. Manhattan plot for UKB GWAS


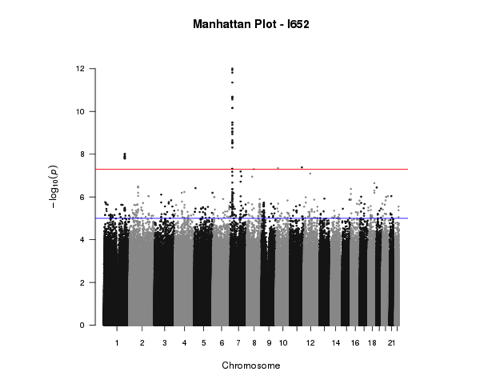


## Table S2. Lead SNVs from UK Biobank CAAD GWAS.

| **rsID** | **Chr** | **Pos (hg19)** | **A1*^a^*** |  | **A2*^a^*** | **MAF** | **BETA** | **SE** | **P** | **eMERGE P** |
| --- | --- | --- | --- | --- | --- | --- | --- | --- | --- | --- |
| rs682112 | 1 | 201746768 | A |  | G | 0.40 | 0.27 | 0.048 | 9.72e-09 | 0.52 |
| rs2526620 | 7 | 19045397 | G |  | A | 0.19 | 0.44 | 0.062 | 9.88e-13 | 0.24 |

1. A1 is the effect and minor allele, A2 the reference allele.

## Figure S3. PheWAS of rs6952610 in eMERGE


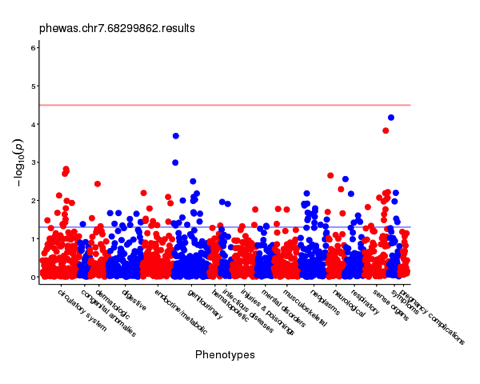


## Table S3. Lowest p-val: Musculoskeletal symptoms referable to limbs symptoms

| Phecode | Description | System | P | OR.CI |
| --- | --- | --- | --- | --- |
| 771 | Musculoskeletal symptoms referable to limbs | symptoms | 6.7e-05 | 1.12 (1.06-1.18) |
| 385 | Other disorders of middle ear and mastoid | sense organs | 1.5e-04 | 1.24 (1.11-1.39) |
| 580.3 | Nephritis and neuropathy with pathological lesion | genitourinary | 2.0e-04 | 1.18 (1.08-1.28) |
| 433.3 | Cerebral ischemia | circulatory system | 1.5e-03 | 1.06 (1.02-1.10) |

#

# Supplemental Methods

# CAAD Phenotype Algorithm Pseudo Code

# 1. Introduction:

Implementation of an electronic medical record (EMR) based phenotype for CAAD within the Electronic Medical Records and Genomics (eMERGE) Network will employ a variety of clinical data types including patient encounter records, diagnoses codes, surgical procedure codes, natural language processing (NLP), laboratory measurements, and medication data.

Phenotype logic is described below beginning in section 6 (General Inclusion Criteria). A flow diagram representing the logic and definitions used to identify CAAD cases and CAAD controls in provided in section 10 as Figure 10.1.

# 2. Development

This phenotype was developed at Kaiser Permanente Washington Health Research Institute/University of Washington (KPWA/UW). Structured data from the KPWA data warehouse and clinical imaging reports from the KPWA EMR were used to define cases and controls. The algorithm defines two categories of cases (severe CAAD and moderate CAAD) and two categories of controls (measured and unmeasured).

KPWA/UW developed a portable NLP system used to extract carotid stenosis exam results from imaging reports. This system was initially shared with developers at Marshfield, Northwestern and Mayo Clinic. Each receiving site processed a sample of reports and conducted error analyses, assisted by the portable NLP system’s built-in diagnostic reporting feature. KPWA/UW iteratively adapted the portable NLP system to address performance issues at each of these sites before releasing final versions of the system. The initial chart review validation was conducted at KPWA/UW. A secondary validation was conducted at Marshfield Clinic (N=40).

# 3. Data Requirements

To identify patients qualifying as cases or controls for this algorithm and to provide covariates needed for analyses the following types of data are needed:

- Patient demographics
- Encounter history
- Free text imaging reports (for natural language processing)
- Diagnosis codes
- Procedure codes
- Medication records

# 4. General Implementation Steps

For efficiency we recommend implementing the CAAD algorithm in the following general steps.

Identify patients meeting criteria described in section 6, General Inclusion Criteria (below).

Assemble clinical text reports of imaging studies that may include assessments of carotid artery stenosis (e.g., carotid ultrasound studies, CT studies of the neck, CS studies of the neck).

Process the assembled clinical text reports using the portable NLP system posted on [PheKB](https://phekb.org) with other CAAD phenotype files, then store the structured results produced by the system (in either CSV format or a database table).

Using your preferred programming language (e.g., SAS) process the NLP-extracted data in the CSV file to identify patients meeting the measured carotid stenosis levels described for cases and controls (sections 7 and 8 below). SAS code for processing the NLP system’s CSV file to produce these patient-level summaries is posted on [PheKB](https://phekb.org) (CAAD_Process_NLP_Data.sas).

Assemble the structured data needed to determine case and control status as described in sections 7 and 8 (below).

Combine the NLP-extracted structured data (Step D above) with other patient data (Step E above) to identify CAAD cases and CAAD controls (sections 7 and 8 below).

Using the data dictionary posted on [PheKB](https://phekb.org), prepare the required datasets of observations and covariates for cases and controls.

As a final “verification” step, randomly select and review the data for two or three of each type of cases and two or three of each type of controls to assure case and control assignments were made using the correct data and logic.

# 5. Using the NLP System

The portable NLP system for extracting results of carotid artery imaging studies can be downloaded from [PheKB](https://phekb.org). A set of fictitious imaging reports useful for testing the NLP system after you install it locally are included in the NLP package. An NLP system User Guide is also available on [PheKB](https://phekb.org).

The NLP system is an easily installed Java application with a simple graphical user interface. The User Guide provides step-by-step user instructions. If you encounter any problems installing or using the NLP system please email or call the contacts listed on page 1.

The NLP system supports two modes for inputting text documents: a) reading the documents as individual files stored in a folder of the local or network file system, or b) reading the documents as text fields from a relational database table. Similarly, output from the NLP system may be stored in either a comma separated value (CSV) format file in the file system or a database table.

As described further in the User Guide, the NLP system generates output at several levels of granularity, each displayed on a separate tab in the user interface. These outputs are intended to simplify local review of results and to facilitate error analysis.

We designed this NLP system to be used as a tool in a remote collaboration between the implementing site (your site) and the developing site (KPWA/UW). KPWA/UW personnel are available to help you identify any errors the NLP system may make when processing your reports, incorporate revisions into the NLP system designed to address these errors, and make available via [PheKB](https://phekb.org) an updated version of the NLP system.

The aim of the NLP system is to create accurate structured data at the level of independent mentions within an imaging study report. For example, from the statement “50-69% stenosis of the left internal carotid artery” the system is designed to extract the minimum reported stenosis value (i.e., 50%) the maximum reported stenosis value (i.e., 69%), and associated document date, document identifier, and patient identifier. These structured data can then be processed by the local programmer along with other structured patient data to implement the phenotype algorithm. The NLP system does not carry out the final step of summarizing the extracted results to the patient level. As noted above, a SAS program (CAAD_Process_NLP_Data.sas) is available on [PheKB](https://phekb.org) to illustrate the process of summarizing the NLP data to the patient level.

# 6. General Inclusion Criteria

All patients must satisfy the following criterion before being considered for inclusion in the study as a case or control.

6.1. In the period preceding the date of the last known contact with the patient (e.g., an outpatient or inpatient encounter) the patient must have evidence of contact that satisfies at least one of the following three criteria:

6.1.1. Contact on at least two (2) occasions ≥365 days apart in the preceding 5 years (e.g., 6/23/2011 and 9/30/2014), OR

6.1.2. Contact in at least three (3) different calendar quarters in the preceding 5 years (e.g., 1/1/2014 and 3/15/2014 and 6/30/2014), OR

6.1.3. Contact in at least four (4) different calendar months in the preceding 5 years (e.g., 9/1/2011 and 10/1/2011 and 11/1/2011 and 12/1/2011).^[[1]](#footnote-1)^

We require this minimum level of contact to assure that patients with CAAD have opportunities for the disease to be documented in their EMR chart. Because risk of CAAD increases with age we require the contact to be within the last five years of the patients most recent known contact with the health care system.

# 7. CAAD Case Criteria

In addition to satisfying the general inclusion criterion (6.1 above), patients may be considered as potential CAAD cases if they:

7.1. Do not have laboratory evidence (ever) of a maximum total cholesterol level >400.

*Note: In the KPWA laboratory system the relevant cholesterol result is labeled as “TOTAL CHOLESTEROL.” Other reported lab results (e.g., for HDL, LDL, and triglycerides) may be ignored at KPWA.*

*CAAD Case Definition #1*

Patients meeting the case eligibility criteria (7.1) will satisfy our CAAD case definition #1 if in addition they:

7.2. Have evidence from a carotid imaging study of >50% carotid artery stenosis (at least unilaterally),

*OR*

7.3. Have at least one procedure code in the medical record indicating the received an endarterectomy (evidenced by an ICD-9 or CPT procedure code). Qualifying procedure codes are:

00.63 (ICD: Percutaneous insertion of carotid artery stent(s))

38.12 (ICD: Endarterectomy of other vessels of head and neck including carotid)

35301 (CPT: Thromboendarterectomy, including patch graft, if performed, carotid …)

35390 (CPT: Reoperation, carotid, thromboendarterectomy, > 1 month after orig. …)

*CAAD Case Definition #2*

Patients meeting the case eligibility criteria (7.1) will satisfy our CAAD case definition #2 if in addition they:

7.4. Have evidence from a carotid imaging study of >-=16% and ≤49% carotid artery stenosis (at least unilaterally),

*OR*

7.5. Have CAAD diagnoses (ICD-9 code 433.1) on at least two occasions ≥30 days apart at any time in the past.

# 8. CAAD Control Criteria

In addition to satisfying the general inclusion criterion (6.1 above), patients may be CAAD controls if they:

8.1. Have evidence from a carotid imaging study of ≤15% carotid artery stenosis *bilaterally* (i.e., there is no evidence of stenosis >15% in either carotid artery),

*OR*

8.2.1 Do not have evidence (ever) of having received an imaging study of the carotid arteries,

*AND*

8.2.2 Do not have any CAAD diagnoses (ICD-9 code 433.1, 433.10, or 433.11),

*AND*

8.2.3 Have never received a carotid repair procedure. Qualifying procedure codes are:

00.63 (ICD: Percutaneous insertion of carotid artery stent(s))

38.12 (ICD: Endarterectomy of other vessels of head and neck including carotid)

35301 (CPT: Thromboendarterectomy, including patch graft, if performed, carotid, …)

35390 (CPT: Reoperation, carotid, thromboendarterectomy, > 1 month after orig. …)

00.61 (ICD: Percutaneous angioplasty or atherectomy of precerebral … vessel(s))

38.02 (ICD: Incision of other vessels of head and neck)

38.62 (ICD: Other excision of other vessels of head and neck)

0075T (CPT: Transcatheter placement of extracranial, vertebral, or … )

0076T (CPT: Transcatheter placement of extracranial, vertebral, or … )

35501 (CPT: Bypass graft, with vein, common carotid-ipsilateral internal carotid …)

33891 (CPT: Bypass graft, with other than vein, transcervical retropharyngeal …)

*Note: The above list includes procedures that do not qualify as carotid repair procedures for purposes of case definition. This is intentional. The above 11 codes optimize sensitivity for detecting possible carotid repair procedures that exclude a patient from being a control; the four codes used to define cases optimize specificity in defining procedures that qualify a patient as a case.*

# 9. KPWA/UW Counts of Cases, Controls, and Excluded Patients

Table 1 shows frequency counts of KPWA/University of Washington patients qualifying as CAAD cases, CAAD controls, or excluded per this CAAD phenotype pseudo. This cohort tends to be older than many other eMERGE cohorts, but the distributions by age group may be informative. Also keep in mind that most KPWA/UW patients receive care through KPWA a managed care model, which may also influence rates of CAAD detection compared to other settings.

| **Table 1. Frequency counts of KPWA/University of Washington patients qualifying as cases, controls, or excluded per the CAAD phenotype pseudo code by age group.** | | | | | | | | |
| --- | --- | --- | --- | --- | --- | --- | --- | --- |
| **Age Group** | **Case Def. #1** | | **Case Def. #2** | | **Control** | | **Exclude** | **Total** |
|  | **Case by 7.2** | **Case by 7.3** | **Case by 7.4** | **Case by 7.5** | **Control by 8.1** | **Control by 8.2** |  |  |
| **50-59** | 0 | 0 | 3 | 1 | 8 | 523 | 64 | 599 |
| **60-69** | 5 | 4 | 9 | 6 | 33 | 1265 | 183 | 1505 |
| **70-79** | 6 | 6 | 21 | 9 | 24 | 672 | 130 | 868 |
| **80-89** | 29 | 30 | 43 | 40 | 70 | 1167 | 418 | 1797 |
| **90+** | 24 | 7 | 37 | 20 | 56 | 657 | 247 | 1048 |
| **All** | 64 | 47 | 113 | 76 | 191 | 4284 | 1042 | 5817 |

# 10. Data Dictionary and Covariates

The CAAD data dictionary with detailed definition of all data elements required for implementing the phenotype is available on [PheKB](https://phekb.org).

Covariates include measures of relevant comorbidities (e.g., cardiovascular disease, diabetes) and medications (e.g., lipid-lowering drugs), smoking status, body mass index, and demographics. Some covariates are measured as patient-level constants or flag variables (e.g., race) and others are captured as repeated measures (e.g., lipid lowering medications). Major categories of covariates described in the data dictionary are:

Patient-level data represented as constants or computed flag variables:

- Patient demographics
- Vital status/follow up status
- Smoking status
- Familial hypercholesterolemia diagnoses
- Type 1 diabetes mellitus (T1DM) diagnoses
- Type 2 diabetes mellitus (T2DM) diagnoses
- Coronary artery disease (CAD) diagnoses
- Peripheral Arterial Disease (PAD) diagnoses
- Endarterectomy (ENDART) procedures
- Cardiac revascularization (CRV) procedures

Repeated measure data:

- BMI
- Diabetes medications
- Lipid lowering medications
- Lipid laboratory results

# 11. Flow diagram

Figure 1 below represents the logic and definitions used to define two categories of CAAD cases and two categories of CAAD controls.


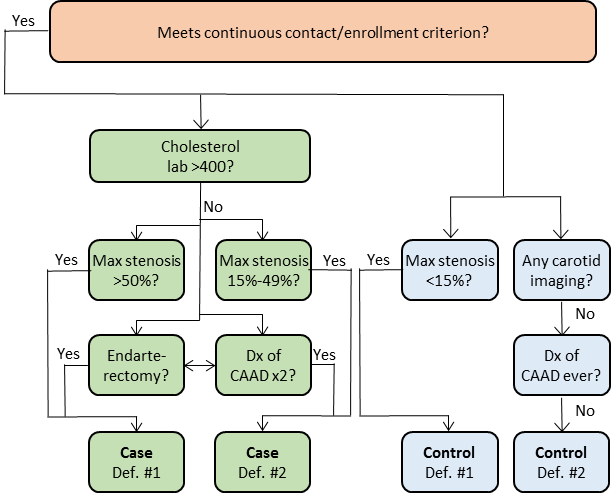


Figure 10.1. Flow diagram of CAAD phenotype logic for definition of cases and controls.

1. SAS code for operationalizing the enrollment/contact criteria using patient encounter data is available upon request from David Carrell, carrell.d@KPWAc.org. [↑](#footnote-ref-1)
